# Automated compartment-based quantification reveals distinct patterns of cutaneous innervation with aging and microbial colonization

**DOI:** 10.64898/2026.10.12.758665

**Published:** 2026-10-13

**Authors:** Henry Le Chang, Max Lee, Lauren Schneider, Élora Midavaine, Beatriz C. Moraes, Crystal A. Chu, Sakeen W. Kashem

## Abstract

Cutaneous innervation is commonly quantified using measures such as intraepidermal nerve fiber density, but these approaches capture only selected anatomical regions or features of nerve structure. We developed an automated image-analysis pipeline to quantify nerve area and nerve-skeleton length across defined epidermal and dermal compartments in whole, stitched mouse plantar skin sections. Automated epidermis–dermis boundary detection showed agreement with manual tracing within the range of inter-observer variation and produced similar downstream measurements. We then applied the pipeline to skin from mice across age and distinct microbial environmental exposures. Conventionalized mice exposed to microbes had greater nerve area and length than germ-free mice in both the epidermis and dermis. In the aging cohort, aged mice had greater dermal nerve area and length than young mice, while epidermal measurements did not differ significantly. We also analyzed the epidermis and dermis together to assess whole-skin innervation. Whole-skin nerve area and length were higher in conventionalized mice than in germ-free mice but did not differ significantly between young and aged mice. These findings show that analyzing the epidermis and dermis separately helps identify where nerve measurements differ, including differences that may not be detected when both skin layers are analyzed together. Compartment-resolved quantification therefore provides a complementary approach for studying changes in cutaneous innervation across biological conditions and behavioral outcomes.

